# Supplementary figures and images for: The potential, analysis and prospect of ctDNA sequencing in hepatocellular carcinoma
Source: PeerJ. 2022 May 17;10:e13473. doi: 10.7717/peerj.13473 (PMC9121877; doi:10.7717/peerj.13473)

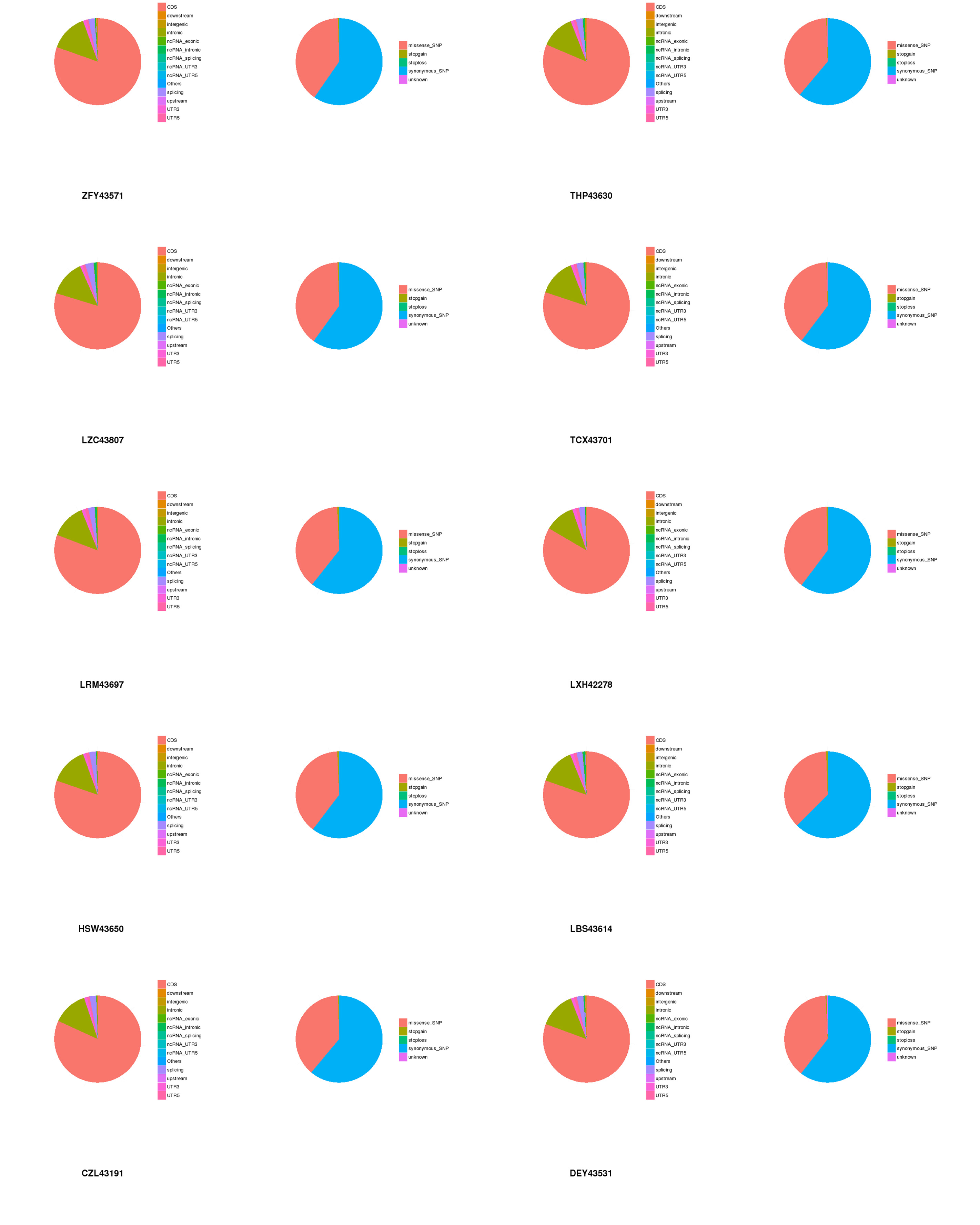

Supplement: Supplemental Information 6 [file peerj-10-13473-s006.jpg]

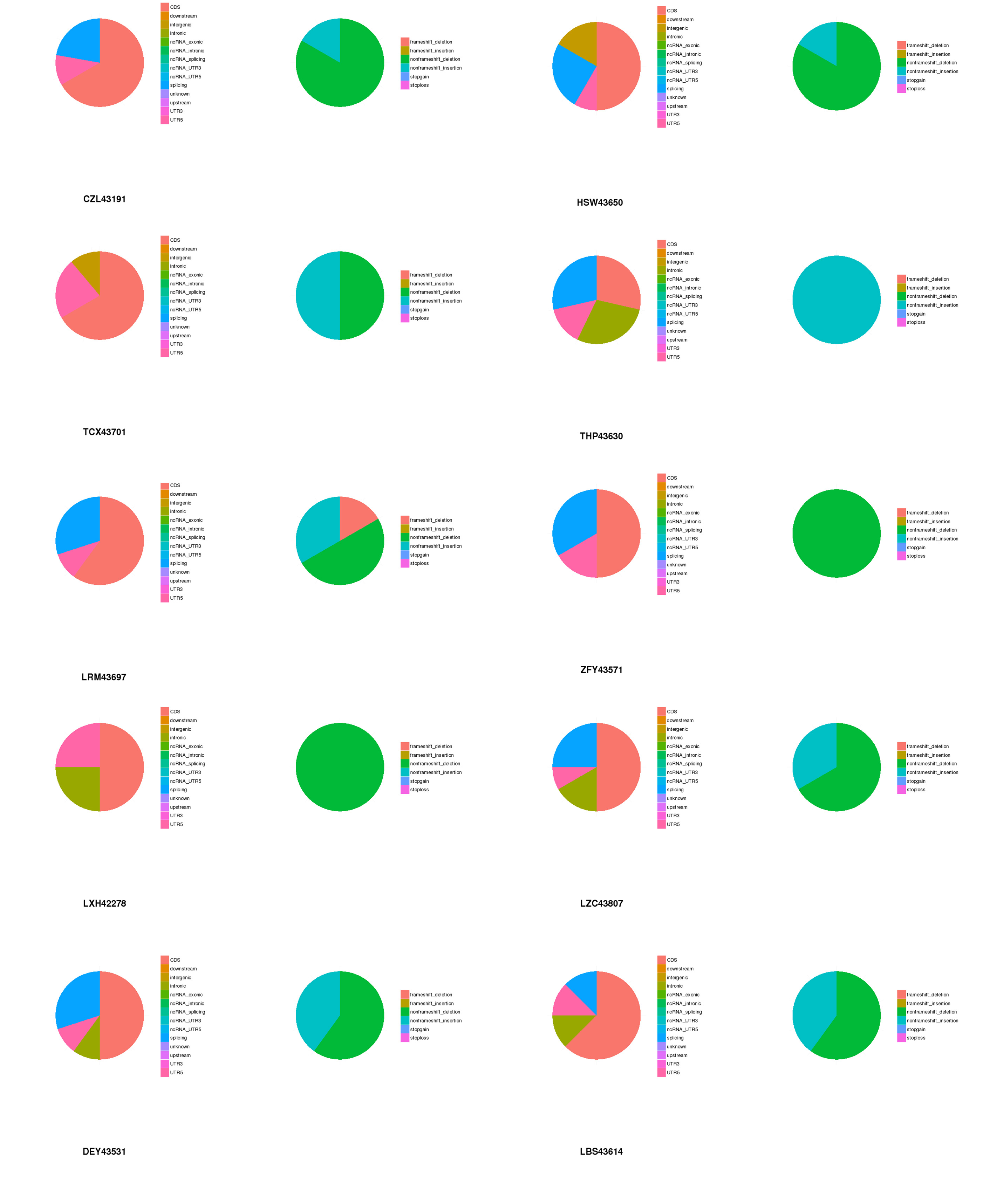

Supplement: Supplemental Information 7 [file peerj-10-13473-s007.jpg]
