# Supplementary material for: The potential, analysis and prospect of ctDNA sequencing in hepatocellular carcinoma
Source: PeerJ. 2022 May 17;10:e13473. doi: 10.7717/peerj.13473 (PMC9121877; doi:10.7717/peerj.13473)
Supplement: Supplemental Information 9 [file peerj-10-13473-s009.zip › Result/Mutation/INDEL/INDEL.Readme (English).pdf]

Insertion/deletion mutation detection

I. Analysis and Result Folder

Description Analysis

Description:

We use samtools for INDEL analysis and ANNOVAR for functional annotation of INDEL.

Explanation of results:

The analysis results are stored in the/Result/Mutation/INDEL folder and contain the following files:

Vcf/*. Indel. Reformated. VCF. GZ: INDEL result file, VCF (http://samtools.github.io/hts-specs/VCFv4.2.pdf) Format

Annotation/*. Indel. Annovar. Hg19 _ multianno. Xls. GZ: The annotation result file of INDEL, which is in xls compressed format and can be used after decompression

Open excel or EditPlus (if file is large)

*. Indel. Pathway. Xls. GZ: Metabolic pathway annotation results of INDEL sites, in xls compressed format. After decompression, you can use excel or EditPlus.

(When the file is large) Open

INDEL _ function. Stat. Xls: Statistical results of different types of INDELs on genomes and coding regions

INdel _ features. Xls: Statistics of INDEL on the genome

II. Introduction to the Header of the Results Document

indel_function.stat.xls

(1) Sample: Sample name

(2) CDS: Exon Coding Region

(3) Frame shift _ deletion: a frameshift deletion that results in the deletion of a change in the reading frame of the encoded protein; The length of the deletion is a non-integer multiple of 3

(4) Frame shift _ insertion: a frameshift insertion that results in a change in the reading frame of the encoded protein; The length of the insertion is a non-integer multiple of 3

(5) Nonframe shift _ deletion: a deletion that is not a frameshift deletion and does not change the reading frame of the encoded protein; The length of the deletion is an integer multiple of 3

(6) Nonframe shift _ insertion: a non-frameshift insertion that does not change the reading frame of the encoded protein; The length of the insertion is an integer multiple of 3

(7) Stopgain: a nonsynonymous mutation that results in a new stop codon at the point of mutation due to an insertion, deletion, or consecutive base substitution

(8) Stoploss: a non-synonymous mutation that results in the loss of a stop codon at a mutation site due to an insertion, deletion, or consecutive base substitution

(9) Unknown: An unknown functional site due to an error in the genetic structure annotation database used for annotation

(10) Intronic: The intronic region of a gene

(11) UTR3: 3'UTR region of the gene

(12) UTR5: 5'UTR region of the gene

(13) UNK1: Splicing site 4 BP region

(14) NcRNA _ exonic: noncoding RNA exon region

(15) NcRNA _ intronic: noncoding RNA intron region

(16) NcRNA _ UTR3: 3'UTR region of non-coding RNA

(17) NcRNA _ UTR5: 5 'UTR region of non-coding RNA

(18) NcRNA _ splicing: 4 BP region of the splicing site of noncoding RNA

(19) Upstream: outside the 1 Kb region upstream of the transcription start site

(20) Down steam: outside the 1 kb region downstream of the transcription termination site

(21) Intergnic: intergenic region

(22) Others: Other area

(23) Total: Total number of INDELs

indel_features.xls

(1) Sample: Sample name

(2) Total: total number of INDELs

(3) Heterozygote genotype

(4) Homozygote

(5) DbSNP percentage: percentage in dbSNP

(6) New: Number of new INDELs not annotated by dbSNP

***.indel.pathway.xls**

(1) CHROM: Chromosome

(2) POS: The locus of variation, which is the absolute position of the chromosome

(3) ID: dbSNP comment ID

(4) REF: Reference genome base pattern

(5) ALT: sample genome base pattern

(6) GeneName: gene name

(7) Pathway Name: metabolic pathway name

(8) Pathway ID: ID of metabolic pathway

(9) Pathway Database

(10) Pathway Description: Description of metabolic pathway

(11) Pathway Link: Metabolic Pathway Web Link

***.indel.annovar.hg19_multianno.xls**

(1) CHROM: Chromosome

(2) POS: The locus of variation, which is the absolute position of the chromosome

(3) ID: dbSNP comment ID

(4) REF: Reference genome base pattern

(5) ALT: sample genome base pattern

(6) QUAL: quality value under Phred standard, indicating the possibility of mutation at this point; The higher the value, the greater the likelihood of a mutation; Calculation method: Phred = -10 * log (1-P); P is the probability of the existence of the mutation.

(7) FILTER: filter TAG, if the site meets all the filter conditions, it is marked as PASS (filter conditions: QUAL > 20; DV

≥4; MQ≥30)

(8) GeneName: a gene name annotation that lists the genes associated with the variant

(9) Func: annotate the region in which the variable site is located (exonic, splicing, UTR5, UTR3, intronic, ncRNA _ exonic, ncRNA _ intronic, nnRNA _ UTR3, ncRNA--UTR5, ncRNA_splicing, upstream, downstream, intergenic)。 Note: 1. Exonic should include coding exonic portion, UTR3 and UTR5, but exonic only represents coding exonic portion in the ANNOVAR annotation result. 2. When a variable site is located in multiple genes or transcripts and has different functions, these functions are sorted according to priority.This column outputs the function type with the highest priority: Exonic = splicing > ncRNA > > UTR5/UTR3 > intron > upstream/down stream > intergenic. When a variant is located in both UTR3 of one gene and UTR5 of another gene, the column outputs "" UTR5, UTR3 "". This column outputs "upstream, down stream" "" when a variant is both downstream of one gene and upstream of another gene

(10) Gene: List the transcripts associated with the variant site (only those transcripts whose functions match the function list are listed). If Func is

Intergenic, where the gene names on both sides are listed

(11) Gene Detail: Describes the variation in the UTR, splicing, ncRNA _ splicing, or intergenic regions. When the value of the Func column is exonic, ncRNA _ exonic, intronic, ncRNA _ intronic, upstream, downstream, upstream; When down stream, ncRNA _ UTR3, ncRNA _ UTR5, the column is empty; When the value of the Func column is intergenic, the column format is dist = 1366; Dist = 22344, representing the distance from the mutation site to the flanking genes

(12) ExonicFunc: SNV or InDel variant types in the exon region (SNV variant types include synonymous _ SNV, missense _ SNV, stopgain _ SNVs, stopgloss _ SNVs, and unknown; Indel variants include frame shift insertion, frameshift deletion, stopgain, stoploss, nonframeshift insertion, Nonframeshift (and unknown)

(13) AA Change: base and amino acid change; If the site is present in multiple transcripts, each transcript is annotated; The format is generally OR4F5: NM _ 001005484: exon1: c.A421G: p.T141A, respectively representing gene name: transcript ID in RefSeq: exon structure: base mutation information in CDNA: amino acid mutation information in protein

(14) Gencode 27: Gencode Annotated Gene Name

(15) WgRna: based on miRBase and snoRNABase, annotate the microRNA and snoRNA related to the variation site, and give Gene names for microRNA and snoRNA

（16） Cytoband: The chromosomal region in which the mutation site is located (observed by Giemas staining). If the variable sites span more than one segment, they are connected by dashes

（17） Target ScanS: UCSC provides the Target ScanS annotation database, which contains microRNA binding sites conserved in the 3'UTR, derived from the prediction results of Target ScanHuman 5.1; The software predicts the target of microRNA, and the prediction results are sorted according to the binding efficiency between microRNA and target. The higher the ranking is, the more likely the binding between microRNA and its target is an actual event. This item gives information about microRNA targets, one is score,It is the score of the target, which reflects the ranking of binding efficacy. Therefore, the higher the score, the lower the ranking, and the lower the possibility of actual binding. The author does not recommend a threshold. The second is Name, which is the name of the microRNA acting on the target. For example, Score = 62; Name = KRAS: miR-181: 1, indicating that the target has a score of 62 and is located in the 3'UTR of the KRAS gene, and the microRNA affected by this variant site is miR-181: 1.Indicates that the variable point is at the binding site of a microRNA (miR-181: 1) on the 3'UTR of the gene KRAS

（18） TfbsConsSites: Based on the transface matrix database (v7.0), calculate the conservative scores of all transcription factor binding sites in the human/mouse/rat alignment. When the score of a binding site reaches a threshold, the site is considered to be conservative in human/mouse/rat. This column gives the position and score of the conserved transcription factor binding site where the variant site is located, i.e. Name and Score.Name is the name of the motif at the binding site, and these motifs can be recognized by transcription factors, for example, V $CDPCR3 _ 01. Some online servers (such as MSigDB) can be used to query which transcription factors can recognize this motif; Score is the conservative score of the binding sit

（19） Genomic SuperDups: Detect whether the mutation site is located in a segmental duplication. Most of the genetic variations detected in duplicated regions are due to sequence alignment errors, so variations annotated into segmental duplications need to be treated with caution and are likely to be false positive sites. Two values are given, one is Name, which indicates the location of a fragment in the genome that is similar to the region in which the variable site is located;The other is Score, which indicates the sequence identity of two similar fragments. For example, Score = 0.994828; Name = chr19: 60000, indicating that the fragment where chr19: 60000 is located is similar to the fragment where the variable site is located, and the sequence identity is 0.994828, ranging from 0 to 1

（20） Repeat: Repeat sequence annotation information. The repeat sequence comes from the RepeatMasker annotation. For example, Score = 180; Name=""1385:(CACCC)n(Simple_repeat)""。 Score represents the score of the repeat; Name consists of two parts, one part (CACCC) n is the name of repeat, and the other part Simple is the type of repeat. As long as there is annotation information, it indicates that the mutation is located in a scattered repetitive sequence or a low-complexity sequence.These regions are prone to alignment errors, so the reliability of variant sites in these regions is not high.

（21） Avsnp150: ID of the variant in dbSNP (version 150)

（22） Cosmic82: annotation of the cosmic database of tumor-associated variants

（23） Clinvar _ 20170905: Annotate the relationship between variation and human health, and the data of clinical significance are from NCBI format: CLINSIG = non-pathogenic; CLNDBN=not_specified; CLNREVSTAT=single; CLNACC=RCV000116259.1; CL NDSDB=MedGen; CLNDSDBID=CN169374。 CLINSIG stands for the clinical significance of the variant site, which can be taken as unknown, untested, non-pathogenic, probable-non-pathogenic, probable-pathogenic, pathogenic, drug-response, histocompatibility, other。 CLINDBN stands for the name of the disease associated with the variant site. CLNREVSTAT represents the verification status of this piece of clinical information, and the values are mult, single, not, exp and Prof. Application of CLNACC

representative variation in CLINVAR database

Number and version number. CLNDSDB is the database source for disease association information, and CLNDSDBID is the number in the database

（24） GwasCatalognew: GWAS (Genome-wide association studies) database annotation

(25) 1000g2015aug _ Chinese: the allele frequency of the mutation base at the mutation site in the Chinese population given the data of the Thousand Genomes Project (version published in August 2015).

(26) 1000g2015aug _ eas: Allele frequency of the mutated base at the mutation site in the East Asian population given the 1000 Genome Project data (version published in August 2015)

(27) 1000g2015aug _ all: Allele frequency of the mutation base at the mutation site in all populations given the 1000 Genome Project data (version published in August 2015)

（28） ESP6500siv2 _ all: Allelic frequency of the mutated base in all individuals with SNP variants, InDel variants, and variants on the Y chromosome in the National Heart, Lung, and Blood Institute Exome Sequencing Project (NHLBI-ESP project, esp6500si _ all database

（29） ExAC _ ALL: ExAC is short for Exome Aggregation Consortium, which integrates data from 60706 unrelated individuals from a large number of disease-specific studies and population genetics studies. Reference set of allele frequency that can be used to study serious diseases. Currently, the ExAC database includes ALL, AFR (African), AMR (Admixed American), EAS (East Asian), FIN (Finnish), NFE (Non-finnish European). OTH (other), SAS (South Asian)。ExAC _ ALL is the allelic frequency of the mutated base at the variable site in all populations

（30） ExAC _ EAS: Allelic frequency of the mutated base at this variant site in the East Asian population of ExAC

（31） SIFT: SIFT score (dbNSFP version 3.0) indicating the effect of the variation on the protein sequence. Before and after the comma are SIFT _ score and SIFT _ pred respectively: SIFT _ score is the SIFT score, and the smaller the score is, the more likely it is to be "harmful", indicating that the SNP is more likely to cause changes in protein structure or function. SIFT _ pred is the prediction result, and the value is T or D. When the variation affects multiple protein sequences at the same time, there is a SIFT value for each protein sequence.Take the minimum value. D: Deleterious (sift<=0.05); T: tolerated (sift>0.05))

（32） Polyphen2 _ HVAR: Polyphen2 was used to predict the effect of this variation on protein sequences based on the HumanVar database for the diagnosis of Mendelian genetic diseases (dbNSFP version 3.0). Polyphen2 _ HVAR _ score and Polyphen2 _ HVAR _ pred are precede and followed by a comma, respectively: Polyphen 2 _ HV _ score is that score of PolyPhen 2, and the high the value, the more likely it is to be "harmful," Indicating that the SNP is more likely to cause changes in protein structure or function; Polyphen2 _ HVAR _ pred is the predicted result, and the value is D or P or B (D: P) (> = 0.909), P:

（33） Polyphen2 _ HDIV: Polyphen2 was used to predict the effect of this variation on protein sequences based on the HumanDiv database for complex

Disease (dbNSFP version 3.0). Polyphen2 _ HDIV _ score and Polyphen2 _ HDIV _ pred are precede and followed by a comma, respectively: Polyphen2HDIVscore is that score of PolyPhen 2, and the high the value, the more likely it is to be "harmful," Indicating that the SNP has high possibility of causing the change of protein structure or function; Polyphen2 _ HDIV _ pred is the predicted result, and the value is D or P or B (D: probably damaging (> = 0.957), P: possibly damaging (0.453 < = pp2 _ hdiv < = 0.956); B: benign (pp2_hdiv<=0.452)

（34） Mutation Taster: Mutation Taster prediction result (dbNSFP version 3.0), indicating the effect of the variation on the protein sequence. Comma

They are Mutation Taster _ score and Mutation Taster _ pred respectively: Mutation taster _ score is the MutationTaster score, ranging from 0 to 1. The higher the score is, the more reliable the prediction result is. Mutation Taster _ pred is the predicted result, which can be A, D, N, or P. "A"" (""Disease_causing_automatic""); ""D"" (""Disease_causing""); ""N"" (""Polymorphism""); ""P"" (""Polymorphism_automatic"")。 D and N are the results of MutationTaster's classification based on score,A and P are obtained using score in combination with other information (if nonsynonymous SNV causes stop-gain, it is predicted to be A; If all three genotypes of nonsynonymous SNV have frequencies in the HapMap, they are predicted to be P). That is to say, MutationTaster's classification results not only depend on the score value, but also combine other information. Therefore, both a and D are deleterious.

(35) Gerp + + GT2: gerp + + in dbNSFP version 3.0 only contains comments for coding variant. To annotate the conservation of all variant sites, ANNOVAR collated gerp + + GT2 to include sites with a GERP + + score greater than 2. The more conservative the site is, the greater the impact on the protein is. The higher the score, the more conservative the locus. In general, sites with a GERP + + score greater than 2 are considered conserved and likely to be functional

(36) CADD: CADD is a tool for scoring the harmfulness of SNV and InDel, which integrates multiple information to annotate the function of variant sites. Not only the functional effects of variations in coding regions (including the effects of synonymous and non-synonymous mutations), but also the functional effects of variations in non-coding regions are predicted. For SNPs, only SNPs in the top 10% of CADD scores are given a score, „. 'Indicates that the CADD score is not in the top 10%.In our annotation results, when there is a score, the commas are CADD and CADD _ Phred respectively; The CADD column is the initial score, and CADD _ Phred is the converted score; When there is no score, that is, a '.', the CADD _ Phred value is less than 10. In the CADD _ Phred score, 10 means that the score ranks in the top 10%, 20 means the top 1%, and 30 means the top 0.1%. Therefore, the lower the score requirement is, the more sites can be retained. For SNPs, the CADD authors recommend a CADD _ Phred score of > 15,Articles usually use 10 or 15; InDel has no recommended values

(37) NovoDb _ WES: Allele frequency of the mutant base at the variant site in the Nuohe normal human exon database

(38) NovoDb _ WGS: Allele frequency of the mutant base at the variant site in the Nuohe normal human genome database

(39) INFO: Information of mutation sites detected by mutation software

(40) FORMAT: Several fields are separated by ":" (corresponding to the right column)

GT: that genotype of the locus (0 represent the same Allele and ref, 1, 2, 3, etc. Represents the different Allele and ref; Pure sum:

0/0,1/1; Heterozygote: 0/1) (Genotype)

PL: standardized genotype likelihood value (corresponding to the three genotypes of 0/0, 0/1 and 1/1, the smaller the value, the better)

DP: that sequence depth of the locus (estimate, filtering pair data with an MQ value of 255 or an error)

DV: Sequencing coverage of non-reference bases at the site (estimate, filtering pairwise data with an MQ value of 255 or error)

SP: P-value likelihood value of chain preference, the larger the value is, the more likely there is chain preference

(41) Samplename: FORMAT information of the sample, corresponding to the FORMAT column

(42) Ori _ REF: The value of the REF column in the VCF file for this site. For InDel, the value of REF in the VCF file may be the same as REF in this file

The value of the column is inconsistent because the InDel site is left-normalized with bcftools norm when using the ANNOVAR annotation.

Causes the REF and ALT in the InDel annotation result file to be shorter than the REF and ALT in the VCF file

(43) Ori _ ALT: The value of the ALT column in the VCF file for the site, i.e., all mutated base types at the site. 0, 1, 2, ... in GT of each sample. The equal numbers are based on the columns Ori _ REF and Ori _ ALT, with 0 being the same as Ori _ REF and 1 being the first mutant base type of Ori _ ALT. The value of the ALT column in this file corresponds to one of the Ori _ ALT columns, but may not be consistent because when using the ANNOVAR annotation,The InDel site was left-normalized with bcftools norm, resulting in the ALT column of InDel having a shorter value than its corresponding ALT in the VCF file

(44) Shared _ Hom: Number of samples with homozygous mutations at the current locus

(45) Shared _ het: Number of samples with heterozygous mutations at the current locus

(46) OMIM: Mendelian Database of Genetic Diseases, giving the names of the genetic diseases associated with the genes in which the loci of variation are located

(47) GWAS _ Pubmed _ pValue: In the previous GWAS study, which article reported that the variant site was associated with the disease, and gave the p-value of the site in the article. The format is: semicolon separated pubmedID (p-value)

(48) HGMD _ ID _ Diseasename: gives the ID of the variant site in the HGMD database and the name of the disease associated with the variant. The format is: semicolon delimited ID _ HGMD (disease _ name)

(49) GO _ BP: Gene Ontology Database Annotation

(50) GO _ CC: Gene Ontology Database Annotation

(51) GO _ MF: Gene Ontology Database Annotation, GO is Gene Ontology Annotation, including Biological Process (BP), Cellular Component (CB), CC) and Molecular Function (MF). Give the name of the biological pathway in which the protein or gene in which the mutation site is located participates

(52) KEGG _ PATHWAY: whole genome and metabolic pathway database annotation, giving the name of the metabolic pathway in which the gene at the mutation site participates

(53) PID _ PATHWAY: Pathway interaction database annotation, giving the name of the pathway that interacts with the protein where the mutation site is located

(54) BIOCARTA _ PATHWAY: BIOCARTA database annotation, giving the name of the molecular pathway in which the gene at the mutation site is involved

(55) REACTOME _ PATHWAY: Human Biological Response and Signaling Pathway Database annotation, giving the name of the signaling pathway in which the gene at the mutation site is involved
