# Supplementary material for: The potential, analysis and prospect of ctDNA sequencing in hepatocellular carcinoma
Source: PeerJ. 2022 May 17;10:e13473. doi: 10.7717/peerj.13473 (PMC9121877; doi:10.7717/peerj.13473)
Supplement: Supplemental Information 9 [file peerj-10-13473-s009.zip › Result/Mutation/INDEL/INDEL.Readme.pdf]

插入缺失变异检测

一、分析及结果文件夹说明

分析说明：

我们使用 samtools 进行 INDEL 分析，用 ANNOVAR 做 INDEL 的功能注释。

结果说明：

分析结果存放于/Result/Mutation/INDEL文件夹中，包含以下文件：

Vcf/*.indel.reformated.vcf.gz ：INDEL 结果文件，vcf（http://samtools.github.io/hts-specs/VCFv4.2.pdf）格式

Annotation/*.indel.annovar.hg19_multianno.xls.gz：INDEL 的注释结果文件，xls 压缩格式，解压后可以用 excel 或 EditPlus（文件较大时）打开

*.indel.pathway.xls.gz：INDEL 位点的代谢通路注释结果，xls 压缩格式，解压后可以用 excel 或 EditPlus（文件较大时）打开

indel_function.stat.xls ：基因组和编码区上不同类型 INDEL 统计结果

indel_features.xls ：基因组上 INDEL 的统计结果

二、结果文件表头介绍

indel_function.stat.xls

（1）Sample：样本名称

（2）CDS：外显子编码区域

（3）frameshift_deletion：移码缺失，导致编码蛋白的读框改变的删除；该删除的长度为非 3 的整数倍

（4）frameshift_insertion：移码插入，导致编码蛋白的读框改变的插入；该插入的长度为非 3 的整数倍

（5）nonframeshift_deletion：非移码缺失，不改变编码蛋白的读框的删除；该删除的长度为 3 的整数倍

（6）nonframeshift_insertion：非移码插入，不改变编码蛋白的读框的插入；该插入的长度为 3 的整数倍

（7）stopgain：非同义突变，由于插入、删除或连续碱基替换导致变异位点处产生一个新的终止密码子

（8）stoploss：非同义突变，由于插入、删除或连续碱基替换导致变异位点处的终止密码子丢失

（9）unknown：由于注释用的基因结构注释数据库的错误导致的未知功能位点

（10）intronic：基因的内含子区域

（11）UTR3：基因的 3'UTR 区域

（12）UTR5：基因的 5'UTR 区域

（13）splicing：剪切位点 4bp 区域

（14）ncRNA_exonic：非编码 RNA 外显子区域

（15）ncRNA_intronic：非编码 RNA 内含子区域

（16）ncRNA_UTR3：非编码 RNA 的 3'UTR 区域

（17）ncRNA_UTR5：非编码 RNA 的 5'UTR 区域

（18）ncRNA_splicing：非编码 RNA 的剪切位点 4bp 区域

（19）upstream：转录起始位点上游 1Kb 区域以外

（20）downsteam：转录终止位点下游 1Kb 区域以外

（21）intergnic：基因间隔区域

（22）Others：其他区域

（23）Total：INDEL 的总数

indel_features.xls

（1）Sample：样本名称

（2）total：INDEL 总数

（3）heterozygote：杂合子基因型

（4）homozygote：纯合子基因型

（5）dbSNP percentage：dbSNP 中的比例

（6）novel：未被 dbSNP 注释的新 INDEL 数目

***.indel.pathway.xls**

（1）CHROM：染色体

（2）POS：变异位点，该位点是染色体的绝对位置

（3）ID：dbSNP 注释 ID

（4）REF：参考基因组碱基型

（5）ALT：样本基因组碱基型

（6）GeneName：基因名称

（7）Pathway Name：代谢通路名称

（8）Pathway ID：代谢通路的 ID

（9）Pathway Database：代谢通路数据库

（10）Pathway Description：代谢通路的描述

（11）Pathway Link：代谢通路的网页链接

***.indel.annovar.hg19_multianno.xls**

（1）CHROM：染色体

（2）POS：变异位点，该位点是染色体的绝对位置

（3）ID：dbSNP 注释 ID

（4）REF：参考基因组碱基型

（5）ALT：样本基因组碱基型

（6）QUAL：Phred 标准下的质量值，表示在该点存在突变的可能性；该值越高，则突变的可能性越大；计算方法：Phred 值=-10*log(1-p)；P 为突变存在的概率

（7）FILTER：过滤 TAG，如果该位点满足所有过滤条件，则标记为 PASS（过滤条件：QUAL≥20；DV≥4；MQ≥30）

（8）GeneName：基因名称注释，列出该变异相关的基因

（9）Func：对变异位点所在的区域进行注释（exonic, splicing, UTR5, UTR3, intronic, ncRNA_exonic, ncRNA_intronic, ncRNA_UTR3, ncRNA_UTR5, ncRNA_splicing, upstream, downstream, intergenic）。说明：1、exonic 应该包括 coding exonic portion、UTR3 和 UTR5，但 ANNOVAR 注释结果中 exonic 只代表 coding exonic portion。2、当一个变异位点位于多个基因或转录本，且功能不同，这些功能按照优先级排序，该列输出优先级最高的功能类型：Exonic = splicing >ncRNA>> UTR5/UTR3 > intron > upstream/downstream > intergenic。当一个变异既位于一个基因的 UTR3，又位于另一个基因的 UTR5 时，该列输出"UTR5,UTR3"。当一个变异既位于一个基因的 downstream，又位于另一个基因的 upstream 时，该列输出" upstream,downstream "

（10）Gene：列出该变异位点相关的转录本（只有功能符合 Func 列的转录本才列出）。如果 Func 为 intergenic，此处列出两侧的基因名

（11）GeneDetail：描述 UTR、splicing、ncRNA_splicing 或 intergenic 区域的变异情况。当 Func 列的值为 exonic、ncRNA_exonic、intronic、ncRNA_intronic、upstream、downstream、upstream;downstream、ncRNA_UTR3、ncRNA_UTR5 时，该列为空；当 Func 列的值为 intergenic 时，该列格式为 dist=1366;dist=22344，表示该变异位点距离两侧基因的距离

（12）ExonicFunc：外显子区的 SNV or InDel 变异类型（SNV 的变异类型包括 synonymous_SNV, missense_SNV, stopgain_SNV, stopgloss_SNV 和 unknown；Indel 的变异类型包括 frameshift insertion, frameshift deletion, stopgain, stoploss, nonframeshift insertion, nonframeshift deletion 和 unknown）

（13）AAChange：碱基和氨基酸改变；如果该位点存在多转录本中，则每个转录本都会进行注释；格式一般为 OR4F5:NM_001005484:exon1:c.A421G:p.T141A，分别代表基因名：RefSeq 中的转录本 ID：外显子结构：CDNA 中碱基突变信息：蛋白中氨基酸突变信息

（14）Gencode27： Gencode 注释的基因名称

（15）wgRna： 基于 miRBase 和 snoRNABase，对变异位点相关的 microRNA 和 snoRNA 进行注释，给出 microRNA 和 snoRNA 的基因名称

（16）cytoband：该变异位点所处的染色体区段（利用 Giemas 染色观察得到的）。如果变异位点跨过多个区段，用短横线连接

（17）targetScanS： UCSC 提供 TargetScanS 注释数据库，库中包含在 3’UTR 中保守的 microRNA 结合位点，来源于 TargetScanHuman 5.1 的预测结果；该软件预测 microRNA 的靶点，预测结果依据 microRNA 与靶点之间结合的效能进行排序，排名越靠前，说明 microRNA 与其靶点的结合越可能是实际存在的事件。此项给出 microRNA 靶点的信息，一是 score，是该靶点的分值，反映的是结合效能的排名，因此，score 越大，说明排名越靠后，实际发生该结合的可能性越小，作者没有推荐阈值；二是 Name，是作用于该靶点的 microRNA 名称。例如，Score=62;Name=KRAS:miR-181:1，表示该靶点的分值是 62，其位于 KRAS 基因的 3’UTR 中，受到该变异位点影响的 microRNA 是 miR-181:1。表示该变异位点位于 microRNA（miR-181:1）在基因 KRAS 的 3’UTR 上的结合位点

（18）tfbsConsSites： 基于 transfac 矩阵数据库（v7.0），计算所有转录因子结合位点在人/小鼠/大鼠比对中的保守分值，当结合位点的分值达到阈值时，认为该位点在人/小鼠/大鼠中保守。该列给出的是该变异位点所在的保守转录因子结合位点的位置和分值，即 Name 和 Score。Name 是结合位点处的 motif 名称，这些 motif 能够被转录因子识别，例如 V$CDPCR3_01，利用一些在线服务器（如 MSigDB）能够查询这个 motif 能够被哪些转录因子识别；Score 是该结合位点的保守分值

（19）genomicSuperDups：检测该变异位点是否位于重复片段（segmental duplication）中。重复区域中检测到的遗传变异大多数是由于序列比对错误造成的，所以被注释到 segmental duplications 的变异需要谨慎对待，很可能是假阳性位点。给出两个值，一是 Name，表示基因组中与该变异位点所在区域相似的片段的位置；二是 Score，表示两个相似片段的序列一致性。例如，Score=0.994828;Name=chr19:60000，表示 chr19:60000 所在片段跟该变异位点所在片段相似，序列一致性为 0.994828，范围 0~1

（20）Repeat：重复序列注释信息，重复序列来源于 RepeatMasker 注释。例如，Score=180;Name="1385:(CACCC)n(Simple_repeat)"。Score 表示该 repeat 的分值；Name 由两部分构成，一部分(CACCC)n 是 repeat 的名称，另一部分 Simple 是 repeat 的类别。只要有注释信息，就表明该变异位于散在重复序列或低复杂度序列中；这些区域容易出现比对错误，所以该区域的变异位点可靠性不高

（21）avsnp150：该变异在 dbSNP（版本 150）中的 ID

（22）cosmic82：cosmic 肿瘤相关变异数据库的注释

（23）clinvar_20170905：注释变异与人类健康之间的关系，临床意义的数据来源于 NCBI 格式为：CLINSIG=non-pathogenic;CLNDBN=not_specified;CLNREVSTAT=single;CLNACC=RCV000116259.1;CLNDSDB=MedGen;CLNDSDBID=CN169374。CLINSIG 代表变异位点在临床意义，可取值为 unknown, untested, non-pathogenic, probable-non-pathogenic, probable-pathogenic, pathogenic, drug-response, histocompatibility, other。CLINDBN 代表变异位点相关的疾病名称。CLNREVSTAT 代表该条临床信息的核查情况，取值为 mult、single、not、exp、prof。CLNACC 代表变异在 CLINVAR 数据库中的 accession

号和版本号。CLNDSDB 是疾病关联信息的数据库来源，CLNDSDBID 是数据库中的编号

（24）gwasCatalognew：GWAS (Genome-wide association studies)数据库注释

（25）1000g2015aug_Chinese： 给出千人基因组计划数据（2015 年 8 月公布的版本）的中国人群中，该变异位点上突变碱基的等位基因频率

（26）1000g2015aug_eas：给出千人基因组计划数据（2015 年 8 月公布的版本）的东亚人群中，该变异位点上突变碱基的等位基因频率

（27）1000g2015aug_all：给出千人基因组计划数据（2015 年 8 月公布的版本）的所有人群中，该变异位点上突变碱基的等位基因频率

（28）esp6500siv2_all：国家心肺和血液研究所外显子组测序计划（NHLBI-ESP project，esp6500si_all 数据库中包含 SNP 变异、InDel 变异和 Y 染色体上的变异的所有个体中，突变碱基的等位基因频率

（29）ExAC_ALL：ExAC 是 Exome Aggregation Consortium 的简称，整合了 60706 个无关个体的数据，这些个体来源于大量 disease-specific 研究和群体遗传学研究，能够用做严重疾病研究的 reference set of allele frequency。目前 ExAC 数据库中包括 ALL, AFR (African), AMR (Admixed American), EAS (East Asian), FIN (Finnish), NFE (Non-finnish European), OTH (other), SAS (South Asian)。ExAC_ALL 是指在所有人群中，该变异位点上突变碱基的等位基因频率

（30）ExAC_EAS：在 ExAC 的东亚人群中，该变异位点上突变碱基的等位基因频率

（31）SIFT：SIFT 分值(dbNSFP version 3.0)，表示该变异对蛋白序列的影响。逗号前后分别是 SIFT_score 和 SIFT_pred：SIFT_score 是 SIFT 分值，分值越小越可能“有害”，表明该 SNP 导致蛋白结构或功能改变的可能性大。SIFT_pred 是预测结果，取值为 T 或者 D。当该变异同时影响多个蛋白序列时，对每条蛋白序列有一个 SIFT 值，取最小值。D: Deleterious (sift<=0.05); T: tolerated (sift>0.05)）

（32）Polyphen2_HVAR：利用 PolyPhen2 基于 HumanVar 数据库预测该变异对蛋白序列的影响，用于孟德尔遗传病的诊断(dbNSFP version 3.0)。逗号前后分别是 Polyphen2_HVAR_score 和 Polyphen2_HVAR_pred：Polyphen2_HVAR_score 是 PolyPhen 2 分值，数值越大越可能“有害”，表明该 SNP 导致蛋白结构或功能改变的可能性大；Polyphen2_HVAR_pred 是预测结果，取值为 D 或 P 或 B（D: Probably damaging (>=0.909), P: possibly damaging (0.447<=pp2_hvar<=0.909); B: benign (pp2_hvar<=0.446)）

（33）Polyphen2_HDIV：利用 PolyPhen2 基于 HumanDiv 数据库预测该变异对蛋白序列的影响，用于复杂疾病(dbNSFP version 3.0)。逗号前后分别是 Polyphen2_HDIV_score 和 Polyphen2_HDIV_pred：Polyphen2_HDIV_score 是 PolyPhen 2 分值，数值越大越可能“有害”，表明该 SNP 导致蛋白结构或功能改变的可能性大；Polyphen2_HDIV_pred 是预测结果，取值为 D 或 P 或 B（D: Probably damaging (>=0.957), P: possibly damaging (0.453<=pp2_hdiv<=0.956); B: benign (pp2_hdiv<=0.452)

（34）MutationTaster：MutationTaster 预测结果(dbNSFP version3.0)，表示该变异对蛋白序列的影响。逗号

前后分别是 MutationTaster_score 和 MutationTaster_pred：MutationTaster_score 是 MutationTaster 分值，取值为 0-1，分值越大，表示预测结果越可靠。MutationTaster_pred 是预测结果，取值为 A、D、N 或者 P 。 "A" ("Disease_causing_automatic"); "D" ("Disease_causing"); "N" ("Polymorphism"); "P" ("Polymorphism_automatic")。D 和 N 是 MutationTaster 根据 score 进行分类的结果，A 和 P 是利用 score 结合其他信息得到的（如果 nonsynonymous SNV 导致 stop-gain，则被预测为 A；如果 nonsynonymous SNV 的三个基因型都在 HapMap 中有频率，则被预测为 P）。也就是说，MutationTaster 的分类结果不仅仅依赖于 score 值，还会结合其它信息。因此，A 和 D 都属于 deleterious。

（35）gerp++gt2：dbNSFP version3.0 中的 gerp++只包含 coding variant 的注释。为了注释所有变异位点的保守性，ANNOVAR 整理了 gerp++gt2，包含 GERP++ 分值大于 2 的位点。越保守的位点发生变异，对于蛋白的影响越大。分值越高，位点越保守。通常，GERP++ 分值大于 2 的位点认为是保守位点，可能具有功能

（36）CADD：CADD 是一种对 SNV、InDel 的有害性进行打分的工具，它整合多种信息来注释变异位点的功能；不仅预测编码区变异（包括同义突变和非同义突变的影响）的功能影响，还预测非编码区变异的功能影响。对于 SNP，仅对 CADD 分值排名在前 10%的 SNP 给出分值，'.'表示 CADD 分值排名不在前 10%。我们的注释结果中，有分值时，逗号前后分别是 CADD 和 CADD_Phred；CADD 列是初始分值，CADD_Phred 是转换后的分值；没有分值，即为'.'时，表示 CADD_Phred 值小于 10。CADD_Phred 分值中，10 表示 score 排名在前 10%，20 表示前 1%，30 表示前 0.1%，因此，分值要求越低，能保留下来的位点越多。对于 SNP，CADD 作者建议 CADD_Phred 分值>15，文章中通常用 10 或 15；InDel 没有建议值

（37）NovoDb_WES： 在诺禾正常人外显子数据库中，该变异位点上突变碱基的等位基因频率

（38）NovoDb_WGS： 在诺禾正常人全基因组数据库中，该变异位点上突变碱基的等位基因频率

（39）INFO：变异软件检测的变异位点信息

（40）FORMAT：用"："分隔了若干个字段(对应右侧一列)

GT：该位点基因型（0 代表 Allele 和 ref 相同，1，2，3 等代表 Allele 和 ref 不同；纯和：0/0，1/1；杂合：0/1）（Genotype）

PL：标准化基因型似然值（对应格式 0/0，0/1，1/1 三种基因型,值越小越好）

DP：该位点测序深度（估计值，过滤了 MQ 值为 255 或者错误的成对数据）

DV：该位点非参考碱基的测序覆盖度（估计值，过滤了 MQ 值为 255 或者错误的成对数据）

SP：链偏好性 P-value 似然值，值越大越可能存在链偏好性

（41）samplename：样本的 FORMAT 信息，与 FORMAT 列一一对应

（42）Ori_REF：该位点在 VCF 文件中 REF 列的值。对于 InDel，VCF 文件中 REF 的值可能跟本文件 REF 列的值不一致，因为使用 ANNOVAR 注释时，用 bcftools norm 对 InDel 位点进行了 left-normalization，

导致 InDel 注释结果文件中的 REF 和 ALT 比 VCF 文件中的 REF、ALT 短

（43）Ori_ALT：该位点在 VCF 文件中 ALT 列的值，即该位点所有的突变碱基型。每个样本的 GT 中 0、1、2、…等编号是依据 Ori_REF 和 Ori_ALT 这两列进行编号的，0 表示跟 Ori_REF 相同，1 表示 Ori_ALT 的第一种突变碱基型。本文件 ALT 列的值对应 Ori_ALT 列中的一个，但可能不一致，因为使用 ANNOVAR 注释时，用 bcftools norm 对 InDel 位点进行了 left-normalization，导致 InDel 的 ALT 列的值比其在 VCF 文件中对应的 ALT 短

（44）shared_hom：在当前位点处发生纯合突变的样本数目

（45）shared_het：在当前位点处发生杂合突变的样本数目

（46）OMIM：孟德尔遗传病数据库注释，给出与变异位点所在基因相关的遗传疾病名称

（47）GWAS_Pubmed_pValue：该变异位点在以往的 GWAS 研究中，被哪篇文章报导与疾病相关联，并给出该位点在文章中的 p-value。格式为：分号分隔的 pubmedID(p-value)

（48）HGMD_ID_Diseasename：给出该变异位点在 HGMD 数据库中的 ID，以及该变异相关的疾病名称。格式为：分号分隔的 ID_HGMD(Disease_name)

（49）GO_BP：Gene Ontology 数据库注释

（50）GO_CC：Gene Ontology 数据库注释

（51）GO_MF：Gene Ontology 数据库注释，GO 是基因本体学注释，包括了基因的生物学过程（Biological Process，BP），细胞组分（Cellular Component，CC）和分子功能（Molecular Function，MF）的注释。给出变异位点所在蛋白质或者基因参与的生物学通路名称

（52）KEGG_PATHWAY：全基因组及代谢途径数据库注释，给出变异位点所在基因参与的代谢通路名称

（53）PID_PATHWAY：通路相互作用数据库注释，给出与变异位点所在蛋白相互作用的通路名称

（54）BIOCARTA_PATHWAY：BIOCARTA 数据库注释，给出变异位点所在基因参与的分子通路名称

（55）REACTOME_PATHWAY：人类生物学反应及信号通路数据库注释，给出变异位点所在基因参与的信号通路名称
