# Supplementary material for: The potential, analysis and prospect of ctDNA sequencing in hepatocellular carcinoma
Source: PeerJ. 2022 May 17;10:e13473. doi: 10.7717/peerj.13473 (PMC9121877; doi:10.7717/peerj.13473)
Supplement: Supplemental Information 9 [file peerj-10-13473-s009.zip › Result/TS.Result_Tree.html]

TS Readme


### TS Readme

Expand all
Collapse all

| Result | Contents | Type |
| --- | --- | --- |
| Mutation | 【Germline突变检测结果】 | folder |
| INDEL | 【indel分析结果】 | folder |
| Annotation | 【注释分析结果】 | folder |
| CZL43191.samtools.indel.annovar.hg19\_multianno.xls.gz | 【INDEL的注释结果文件,xls压缩格式,解压后可以用excel或EditPlus(文件较大时)打开】 | file |
| CZL43191.samtools.indel.pathway.xls.gz | 【INDEL位点的代谢通路注释结果,xls压缩格式,解压后可以用excel或EditPlus(文件较大时)打开】 | file |
| DEY43531.samtools.indel.annovar.hg19\_multianno.xls.gz | 【INDEL的注释结果文件,xls压缩格式,解压后可以用excel或EditPlus(文件较大时)打开】 | file |
| DEY43531.samtools.indel.pathway.xls.gz | 【INDEL位点的代谢通路注释结果,xls压缩格式,解压后可以用excel或EditPlus(文件较大时)打开】 | file |
| HSW43650.samtools.indel.annovar.hg19\_multianno.xls.gz | 【INDEL的注释结果文件,xls压缩格式,解压后可以用excel或EditPlus(文件较大时)打开】 | file |
| HSW43650.samtools.indel.pathway.xls.gz | 【INDEL位点的代谢通路注释结果,xls压缩格式,解压后可以用excel或EditPlus(文件较大时)打开】 | file |
| LBS43614.samtools.indel.annovar.hg19\_multianno.xls.gz | 【INDEL的注释结果文件,xls压缩格式,解压后可以用excel或EditPlus(文件较大时)打开】 | file |
| LBS43614.samtools.indel.pathway.xls.gz | 【INDEL位点的代谢通路注释结果,xls压缩格式,解压后可以用excel或EditPlus(文件较大时)打开】 | file |
| LRM43697.samtools.indel.annovar.hg19\_multianno.xls.gz | 【INDEL的注释结果文件,xls压缩格式,解压后可以用excel或EditPlus(文件较大时)打开】 | file |
| LRM43697.samtools.indel.pathway.xls.gz | 【INDEL位点的代谢通路注释结果,xls压缩格式,解压后可以用excel或EditPlus(文件较大时)打开】 | file |
| LXH42278.samtools.indel.annovar.hg19\_multianno.xls.gz | 【INDEL的注释结果文件,xls压缩格式,解压后可以用excel或EditPlus(文件较大时)打开】 | file |
| LXH42278.samtools.indel.pathway.xls.gz | 【INDEL位点的代谢通路注释结果,xls压缩格式,解压后可以用excel或EditPlus(文件较大时)打开】 | file |
| LZC43807.samtools.indel.annovar.hg19\_multianno.xls.gz | 【INDEL的注释结果文件,xls压缩格式,解压后可以用excel或EditPlus(文件较大时)打开】 | file |
| LZC43807.samtools.indel.pathway.xls.gz | 【INDEL位点的代谢通路注释结果,xls压缩格式,解压后可以用excel或EditPlus(文件较大时)打开】 | file |
| TCX43701.samtools.indel.annovar.hg19\_multianno.xls.gz | 【INDEL的注释结果文件,xls压缩格式,解压后可以用excel或EditPlus(文件较大时)打开】 | file |
| TCX43701.samtools.indel.pathway.xls.gz | 【INDEL位点的代谢通路注释结果,xls压缩格式,解压后可以用excel或EditPlus(文件较大时)打开】 | file |
| THP43630.samtools.indel.annovar.hg19\_multianno.xls.gz | 【INDEL的注释结果文件,xls压缩格式,解压后可以用excel或EditPlus(文件较大时)打开】 | file |
| THP43630.samtools.indel.pathway.xls.gz | 【INDEL位点的代谢通路注释结果,xls压缩格式,解压后可以用excel或EditPlus(文件较大时)打开】 | file |
| ZFY43571.samtools.indel.annovar.hg19\_multianno.xls.gz | 【INDEL的注释结果文件,xls压缩格式,解压后可以用excel或EditPlus(文件较大时)打开】 | file |
| ZFY43571.samtools.indel.pathway.xls.gz | 【INDEL位点的代谢通路注释结果,xls压缩格式,解压后可以用excel或EditPlus(文件较大时)打开】 | file |
| indel\_features.xls | 【基因组上INDEL的统计结果 】 | table |
| indel\_function.stat.xls | 【基因组和编码区上不同类型INDEL统计结果】 | table |
| INDEL.Readme.pdf | 【indel分析结果，pdf格式】 | figure |
| Vcf | 【VCF格式文件】 | folder |
| CZL43191.samtools.indel.reformated.vcf.gz | 【INDEL结果文件,vcf(http//samtools.github.io/hts-specs/VCFv4.2.pdf)格式】 | file |
| DEY43531.samtools.indel.reformated.vcf.gz | 【INDEL结果文件,vcf(http//samtools.github.io/hts-specs/VCFv4.2.pdf)格式】 | file |
| HSW43650.samtools.indel.reformated.vcf.gz | 【INDEL结果文件,vcf(http//samtools.github.io/hts-specs/VCFv4.2.pdf)格式】 | file |
| LBS43614.samtools.indel.reformated.vcf.gz | 【INDEL结果文件,vcf(http//samtools.github.io/hts-specs/VCFv4.2.pdf)格式】 | file |
| LRM43697.samtools.indel.reformated.vcf.gz | 【INDEL结果文件,vcf(http//samtools.github.io/hts-specs/VCFv4.2.pdf)格式】 | file |
| LXH42278.samtools.indel.reformated.vcf.gz | 【INDEL结果文件,vcf(http//samtools.github.io/hts-specs/VCFv4.2.pdf)格式】 | file |
| LZC43807.samtools.indel.reformated.vcf.gz | 【INDEL结果文件,vcf(http//samtools.github.io/hts-specs/VCFv4.2.pdf)格式】 | file |
| TCX43701.samtools.indel.reformated.vcf.gz | 【INDEL结果文件,vcf(http//samtools.github.io/hts-specs/VCFv4.2.pdf)格式】 | file |
| THP43630.samtools.indel.reformated.vcf.gz | 【INDEL结果文件,vcf(http//samtools.github.io/hts-specs/VCFv4.2.pdf)格式】 | file |
| ZFY43571.samtools.indel.reformated.vcf.gz | 【INDEL结果文件,vcf(http//samtools.github.io/hts-specs/VCFv4.2.pdf)格式】 | file |
| SNP | 【SNP突变结果】 | folder |
| Annotation | 【注释分析结果】 | folder |
| CZL43191.samtools.snp.annovar.hg19\_multianno.xls.gz | 【SNP的注释结果文件,xls压缩格式,解压后可以用excel或EditPlus(文件较大时)打开】 | file |
| CZL43191.samtools.snp.pathway.xls.gz | 【SNP位点的代谢通路注释结果文件,xls压缩格式,解压后可以用excel或EditPlus(文件较大时)打开】 | file |
| DEY43531.samtools.snp.annovar.hg19\_multianno.xls.gz | 【SNP的注释结果文件,xls压缩格式,解压后可以用excel或EditPlus(文件较大时)打开】 | file |
| DEY43531.samtools.snp.pathway.xls.gz | 【SNP位点的代谢通路注释结果文件,xls压缩格式,解压后可以用excel或EditPlus(文件较大时)打开】 | file |
| HSW43650.samtools.snp.annovar.hg19\_multianno.xls.gz | 【SNP的注释结果文件,xls压缩格式,解压后可以用excel或EditPlus(文件较大时)打开】 | file |
| HSW43650.samtools.snp.pathway.xls.gz | 【SNP位点的代谢通路注释结果文件,xls压缩格式,解压后可以用excel或EditPlus(文件较大时)打开】 | file |
| LBS43614.samtools.snp.annovar.hg19\_multianno.xls.gz | 【SNP的注释结果文件,xls压缩格式,解压后可以用excel或EditPlus(文件较大时)打开】 | file |
| LBS43614.samtools.snp.pathway.xls.gz | 【SNP位点的代谢通路注释结果文件,xls压缩格式,解压后可以用excel或EditPlus(文件较大时)打开】 | file |
| LRM43697.samtools.snp.annovar.hg19\_multianno.xls.gz | 【SNP的注释结果文件,xls压缩格式,解压后可以用excel或EditPlus(文件较大时)打开】 | file |
| LRM43697.samtools.snp.pathway.xls.gz | 【SNP位点的代谢通路注释结果文件,xls压缩格式,解压后可以用excel或EditPlus(文件较大时)打开】 | file |
| LXH42278.samtools.snp.annovar.hg19\_multianno.xls.gz | 【SNP的注释结果文件,xls压缩格式,解压后可以用excel或EditPlus(文件较大时)打开】 | file |
| LXH42278.samtools.snp.pathway.xls.gz | 【SNP位点的代谢通路注释结果文件,xls压缩格式,解压后可以用excel或EditPlus(文件较大时)打开】 | file |
| LZC43807.samtools.snp.annovar.hg19\_multianno.xls.gz | 【SNP的注释结果文件,xls压缩格式,解压后可以用excel或EditPlus(文件较大时)打开】 | file |
| LZC43807.samtools.snp.pathway.xls.gz | 【SNP位点的代谢通路注释结果文件,xls压缩格式,解压后可以用excel或EditPlus(文件较大时)打开】 | file |
| TCX43701.samtools.snp.annovar.hg19\_multianno.xls.gz | 【SNP的注释结果文件,xls压缩格式,解压后可以用excel或EditPlus(文件较大时)打开】 | file |
| TCX43701.samtools.snp.pathway.xls.gz | 【SNP位点的代谢通路注释结果文件,xls压缩格式,解压后可以用excel或EditPlus(文件较大时)打开】 | file |
| THP43630.samtools.snp.annovar.hg19\_multianno.xls.gz | 【SNP的注释结果文件,xls压缩格式,解压后可以用excel或EditPlus(文件较大时)打开】 | file |
| THP43630.samtools.snp.pathway.xls.gz | 【SNP位点的代谢通路注释结果文件,xls压缩格式,解压后可以用excel或EditPlus(文件较大时)打开】 | file |
| ZFY43571.samtools.snp.annovar.hg19\_multianno.xls.gz | 【SNP的注释结果文件,xls压缩格式,解压后可以用excel或EditPlus(文件较大时)打开】 | file |
| ZFY43571.samtools.snp.pathway.xls.gz | 【SNP位点的代谢通路注释结果文件,xls压缩格式,解压后可以用excel或EditPlus(文件较大时)打开】 | file |
| snp\_features.xls | 【基因组上SNP的统计结果,xls格式 】 | table |
| snp\_function.stat.xls | 【基因组和编码区不同区域上SNP的数目统计结果,xls格式】 | table |
| SNP.Readme.pdf | 【SNP突变结果，pdf格式】 | figure |
| Vcf | 【VCF格式文件】 | folder |
| CZL43191.samtools.snp.reformated.vcf.gz | 【INDEL结果文件,vcf(http//samtools.github.io/hts-specs/VCFv4.2.pdf)格式】 | file |
| DEY43531.samtools.snp.reformated.vcf.gz | 【INDEL结果文件,vcf(http//samtools.github.io/hts-specs/VCFv4.2.pdf)格式】 | file |
| HSW43650.samtools.snp.reformated.vcf.gz | 【INDEL结果文件,vcf(http//samtools.github.io/hts-specs/VCFv4.2.pdf)格式】 | file |
| LBS43614.samtools.snp.reformated.vcf.gz | 【INDEL结果文件,vcf(http//samtools.github.io/hts-specs/VCFv4.2.pdf)格式】 | file |
| LRM43697.samtools.snp.reformated.vcf.gz | 【INDEL结果文件,vcf(http//samtools.github.io/hts-specs/VCFv4.2.pdf)格式】 | file |
| LXH42278.samtools.snp.reformated.vcf.gz | 【INDEL结果文件,vcf(http//samtools.github.io/hts-specs/VCFv4.2.pdf)格式】 | file |
| LZC43807.samtools.snp.reformated.vcf.gz | 【INDEL结果文件,vcf(http//samtools.github.io/hts-specs/VCFv4.2.pdf)格式】 | file |
| TCX43701.samtools.snp.reformated.vcf.gz | 【INDEL结果文件,vcf(http//samtools.github.io/hts-specs/VCFv4.2.pdf)格式】 | file |
| THP43630.samtools.snp.reformated.vcf.gz | 【INDEL结果文件,vcf(http//samtools.github.io/hts-specs/VCFv4.2.pdf)格式】 | file |
| ZFY43571.samtools.snp.reformated.vcf.gz | 【INDEL结果文件,vcf(http//samtools.github.io/hts-specs/VCFv4.2.pdf)格式】 | file |

Tip:

- 该文件为此项目结果文件所有结果的简要说明，所有文件名点击均可链接到相应的文件；为保证链接正常，请保持其文件位置位于结果文件根目录下。


---

如有疑问请咨询项目销售或运营，我们将竭诚为您服务。感谢您对Novogene的信任！ |
